# Henoch-Schonlein Purpura—A Case Report and Review of the Literature

**DOI:** 10.1155/2010/597648

**Published:** 2010-05-23

**Authors:** Amit B. Sohagia, Srinivas Guptha Gunturu, Tommy R. Tong, Hilary I. Hertan

**Affiliations:** ^1^Gastroenterology, Montefiore Medical Center, North Division, 600, E 233rd street, Bronx, NY 10466, USA; ^2^Internal Medicine, Montefiore Medical Center, North Division, 600, E 233rd street, Bronx, NY 10466, USA; ^3^Department of Pathology, Montefiore Medical Center, North Division, 600, E 233rd street, Bronx, NY 10466, USA; ^4^Clinical Medicine, New York Medical College, Valhalla, NY 10595, USA

## Abstract

We describe a case of an adolescent male with Henoch-Schonlein purpura (HSP), presenting with cutaneous and gastrointestinal manifestations. Endoscopy revealed diffuse ulcerations in the stomach, duodenum, and right colon. Biopsies revealed a leukocytoclastic vasculitis in the skin and gastrointestinal tract. Steroid therapy led to complete resolution of the symptoms. HSP is the most common childhood vasculitis, and is characterized by the classic tetrad of nonthrombocytopenic palpable purpura, arthritis or arthralgias, gastrointestinal and renal involvement. It is a systemic disease where antigen-antibody (IgA) complexes activate the alternate complement pathway, resulting in inflammation and small vessel vasculitis. Mild disease resolves spontaneously, and symptomatic treatment alone is sufficient. Systemic steroids are recommended for moderate to severe HSP. The prognosis depends upon the extent of renal involvement, which requires close followup. Early recognition of multiorgan involvement, especially outside of the typical age group, as in our adolescent patient, and appropriate intervention can mitigate the disease and limit organ damage.

## 1. Henoch-Schonlein Purpura

### 1.1. Case

**Figure 1:**
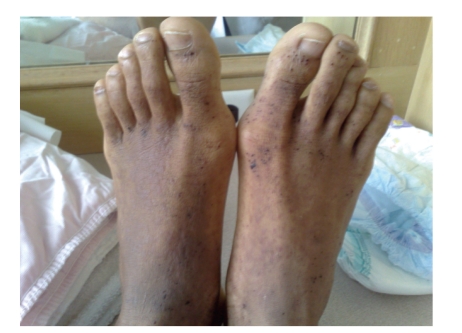
Clinical picture of palpable purpura involvement of bilateral lower extremities.

**Figure 2:**
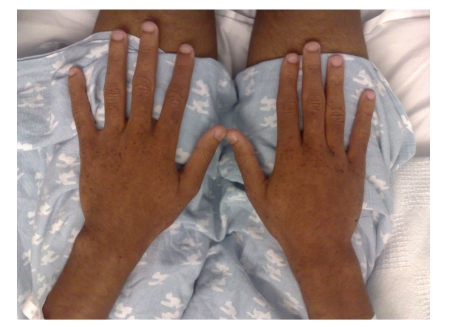
Clinical picture of palpable purpura involvement of bilateral upper extremities.

**Figure 3:**
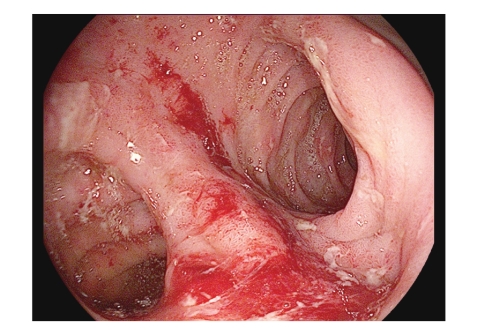
Endoscopic finding on EGD showing inflammation, submucosal hemorrhage, and small ulceration.

**Figure 4:**
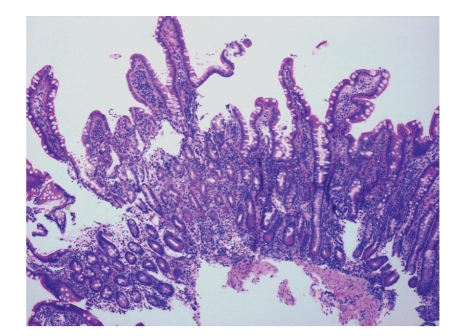
Small bowel biopsy showing preserved villous architecture.

**Figure 5:**
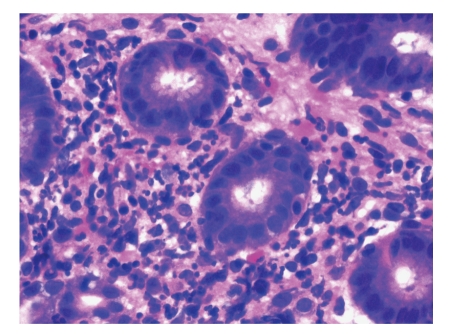
Histopathology of HSP involvement in small bowel showing neutrophilic and eosinophilic infiltrates which are seen with karyorrhectic debris.

**Figure 6:**
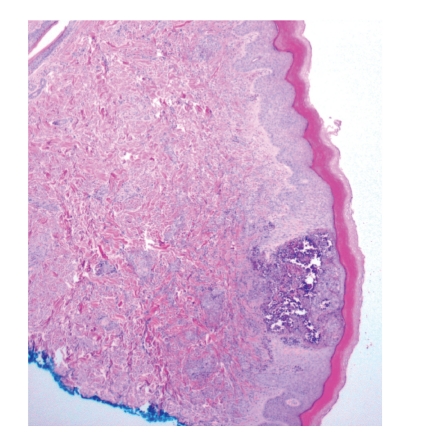
Histopathology of HSP involvement in skin showing microabscess.

A 16-year-old Hispanic male from Puerto Rico presented with fever and sore throat for 5 days. He was given oral penicillin by his primary care doctor. After a day, he developed an erythematous, nonpruritic rash which progressed proximally from both feet to thighs and upper extremities including palms and soles. Later the feet became swollen with moderately intense (7/10) burning pain, aggravated by ambulation. Review of systems is unremarkable except left ear pain. His significant comorbidities include asthma and gastrojejunostomy following a remote motor vehicle accident. On physical examination, there was pharyngeal erythema, petechiae on the soft palate, cervical lymphadenopathy, a nodular, and nontender, nonblanching purpuric rash involving both upper and lower extremities with nonpitting pedal edema (Figures 1 and 2). There was no truncal involvement. Two days later, the patient developed abdominal pain involving the right and left upper quadrant which was constant, colicky in nature, 8/10 in intensity, aggravated with meals, and associated with hematemesis and watery stools. Laboratory tests showed leukocytosis (WBC: 16,900/microL); Hb: 14 g/dL; Hct: 41.2%; BUN: 16 mg/dL; Serum Creatinine: 0.9 mg/dL; Urinalysis: no hematuria or proteinuria; ESR: 58 mm/Hr; CRP: 5.6 mg/dL; Antistreptolysin O titer: 823 IU/L; C3: 125 mg/dL; C4: 11 mg/dL; ANA: Negative; EBV-VCA IgM: Negative; Anti-HAV IgM: Negative; HbsAg: Negative; Anti-HBc IgG: Negative; Mono spot test: Negative; c-ANCA: 0.2 units; p-ANCA: 0.2 units; Stool for occult blood: positive. Esophagogastroduodenoscopy (Figure 3) showed multiple erosions in the duodenum, antrum, and the gastrojejunal anastomotic site. Histopathology (Figures 4 and 5) of the small bowel showed preserved villous architecture, and neutrophilic and eosinophilic infiltrates with leukocytoclastic vasculitis. Warthin-Starry stain was negative for Helicobacter pylori. There was no evidence of epithelioid granuloma. Colonoscopy showed erythema and inflammation in the terminal ileum and cecum. Cecal biopsy also showed leukocytoclastic vasculitis. Skin biopsy (Figure 6) showed pustular leukocytoclastic fibrinoid vasculitis with microabscess. The patient was diagnosed with Henoch-Schonlein purpura as per American College of Rheumatology and European League Against Rheumatism (EuLAR) and Pediatric Rheumatology Society (PReS) criteria. He was treated with intravenous fluids and kept nothing by mouth for 3 days with no enteral or parenteral nutrition. He was started on oral prednisone 20 mg twice a day, with resolution of his symptoms and a decrease in ESR and CRP. His diet was advanced to regular diet on the fourth hospital day.

## 2. Review of Literature

### 2.1. Introduction

Henoch-Schonlein purpura (HSP) is a self-limited, systemic, nongranulomatous, autoimmune complex, small vessel vasculitis, with multiorgan involvement. Its etiology is unclear but is associated with infections (bacterial, viral, parasitic), medications, vaccination, tumors (non-small cell lung cancer, prostate cancer, and hematological malignancies), alpha-1-antitrypsin deficiency, and Familial Mediteranean Fever [1]. (Table 1). HSP is the most common cutaneous vasculitis in children comprising up to 90% of cases [1–3].

### 2.2. Epidemiology

The annual incidence varies geographically from 6.2 to 70.3 per 100,000 in children less than 17 years of age with slight male predominance (M : F = 1.2 : 1.0). Peak age incidence is 4–6 years and 90% of HSP cases occur before the age of 10 years. Worldwide, Afro-Caribbeans have the least incidence while Asians have the highest incidence. In North America, the incidence is 13.5 per 100,000 children and Caucasians have the highest incidence while Afro-Americans have the lowest incidence. HSP is most commonly seen in winter and spring seasons. In adults, the incidence varies between 3.4–14.3 per million population. As this disease is self limited, its true incidence may be underreported [4, 5].

### 2.3. Pathophysiology

**Figure 7:**
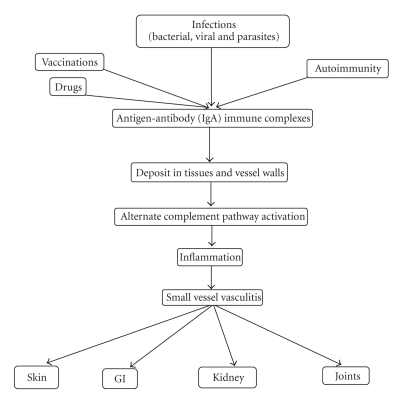
Schematic diagram of HSP pathophysiology.

Antigen and antibody complexes, mostly IgA, form as a result of bacterial and viral infections, vaccinations, drugs, and autoimmune mechanisms [3]. These antigen antibody complexes deposit in the small vessel walls and activate the alternate complement pathway which leads to neutrophil accumulation resulting in inflammation and vasculitis without a granulomatous reaction. This can involve multiple systems including skin, gastrointestinal tract, kidney, and joints but it can involve any organ system. Vasculitis causes extravasation of blood and its components into the interstitial spaces resulting in edema and hemorrhage. (Figure 7) In our case with high ASO titers, streptococcal infection might possibly have played a role in initiating the HSP cascade.

### 2.4. Clinical Features

HSP is characterized by a classic tetrad of nonthrombocytopenic palpable purpura, arthritis or arthralgias, gastrointestinal and renal involvement, and rarely, other systems (lungs, central nervous system, genitourinary tract) [2, 4, 6, 7]. Cutaneous involvement is the most common presentation, although patients may present with involvement of other organ systems.

#### 2.4.1. Skin

Cutaneous manifestations include nonthrombocytopenic rash which evolves from erythematous to urticarial and macular wheels to nonblanching palpable purpura with petechiae and ecchymoses. Palpable purpura is seen in 50% of the cases as the presenting sign. Purpuric lesions occur in groups and may persist up to 3–10 days. Classical HSP is symmetrical in distribution involving dependent areas such as the lower extremities and buttocks but it can also be seen in the upper extremities (Figures 2 and 3). Truncal and facial involvement can also be seen. Initially the lesions are single and less than 1 cm, but later coalesce to form ecchymotic areas. Rarely, hemorrhagic bullae, ulcerations or dermal scarring may be seen. On histopathology (Figures 4 and 5) leukocytoclastic vasculitis, characterized by neutrophilic infiltration and prominent nuclear fragmentation, involving the upper and middle layers of the dermis with IgA deposition on immunofluorescence, is seen. Angioedema (nonpitting edema) can be seen in the scalp, back, and extremities.

#### 2.4.2. Gastrointestinal (GI)

Abdominal pain (colicky in nature, worse with food) is the most common symptom of gastrointestinal involvement. Other symptoms include nausea, vomiting, hematemesis, melena, and hematochezia. These symptoms are secondary to vasculitis involving the splanchnic circulation (mesenteric vasculitis). Rarely, intussuception (ileoleal), ischemic necrosis of the bowel wall, intestinal perforation, massive gastrointestinal bleeding, acute acalculous cholecystitis, hemorrhagic ascites with serositis, pancreatitis, and biliary cirrhosis may occur. Usually, skin manifestations precede gastrointestinal manifestations, but in one fourth of the cases skin lesions occur after gastrointestinal manifestations. HSP should be considered in the differential diagnosis of an acute abdomen (especially in children). Endoscopic findings include erythema, edema, petechiae, ulcers, nodular changes, hematoma-like protrusions, skip hyperemic ecchymotic lesions, and strictures. These are seen in the gastric antrum, cecum, ileum, and colon. The ulcers are small, less than 1 cm^2^, superficial, multiple, irregular, and clean-based. The second portion of the duodenum is most commonly involved. On colonoscopy, the ulcers can be as large as 2 cm^2^, and are most common in the rectum and ileum. The lesions in the ileum are more severe in presentation than the other areas of involvement.

#### 2.4.3. Joints

Joint involvement is seen in up to two-thirds of HSP patients. In one fourth of HSP patients, this can be the presenting sign. Typically, nonmigratory, nondestructive polyarthralgias occur which are symmetrical in distribution and mostly involve the knees and ankles. Joint involvement is more commonly seen in adults than in children.

#### 2.4.4. Renal

Hematuria (microscopic or gross) is the most common renal manifestation. Proteinuria is either seen along with hematuria (commonly) or isolated (rarely). Most of the cases of HSP nephritis resolve spontaneously, only 5% progress to chronic end-stage renal disease (ESRD) at 5 years. Renal involvement is similar to IgA nephropathy (gross hematuria and mild proteinuria following an upper respiratory tract infection). Persistent proteinuria and hematuria predict the development of ESRD. Although most of the patients develop renal involvement within 3 months of skin manifestations, they should be followed for a year with urinalysis. Renal involvement is the most important prognostic factor in determining morbidity and mortality from HSP.

### 2.5. Diagnosis

HSP is a clinical diagnosis but when the presentation is atypical, tissue biopsy may be helpful [8, 9]. Although new criteria are proposed by the European League Against Rheumatism (EuLAR) and Pediatric Rheumatology Society (PReS), most of the studies used old criteria proposed by American College of Rheumatology. For these criteria, please refer to Table 2.

### 2.6. Differential Diagnosis

Children (less than 17 years of age) presenting with palpable purpura and multisystem involvement (GI, kidney and joints) without thrombocytopenia may be diagnosed as HSP. The differential diagnosis of HSP includes conditions such as Crohn's disease, Wegener's granulomatosis, infective endocarditis, IgA nephropathy, and hemolytic uremic syndrome. Hypersensitivity vasculitis can present as leukocytoclastic vasculitis involving the skin (palpable purpura) and rarely, the gastrointestinal tract, but unlike HSP, IgA deposition is not seen. In Crohn's disease and IgA nephropathy, there is no palpable purpura.

### 2.7. Treatment

Treatment of HSP reflects its self-limiting nature in 94% of children and 89% of adults. Symptomatic treatment will be sufficient for symptoms such as rash and arthritis. Acetaminophen and nonsteroidal antiinflammatory drugs can be used [4]. Aspirin should be avoided in children.

Oral steroids are indicated in patients with severe rash, edema, severe colicky abdominal pain (without nausea, vomiting), renal, scrotal, and testicular involvement. Usually prednisone or methylprednisolone can be started at 1 to 2 mg/kg per day for one to two weeks, tapering down to 0.5 mg/kg/day over the next week and then 0.5 mg/kg every other day for one more week. Intravenous (IV) steroids can be administered if the patient does not tolerate oral steroids. Early steroid therapy decreases gastrointestinal symptoms within 2 days compared to 12.3 days in patients without steroids and may decrease HSP or gastrointestinal recurrence and reduce renal progression. Steroids may prevent major complications such as gastrointestinal bleeding or intussusception [10, 13–15]. High-dose IV pulse steroids are indicated in patients with nephrotic range proteinuria and mesenteric vasculitis. These pulse doses can range from 500 mg to 1 gm with various protocols leading to complete remission of nephritis in a few studies [13]. All patients with severe renal involvement should be referred to the nephrologist and a renal biopsy is recommended.

Immunosuppressive drugs (cyclophosphamide, azathioprine, cyclosporine A, and mycophenolate mofetil) in combination with high-dose IV pulse steroids are recommended if there is no benefit from steroids alone. This is usually recommended in rapidly progressive glomerulonephritis (RPGN) and hemorrhagic involvement of the lungs and brain. However, not all studies can confirm the benefits of treatment with steroids and immunosuppressive drugs in preventing kidney involvement compared to placebo groups [11, 16–18] (Table 3).

Plasmapheresis or high-dose intravenous immunoglobulin therapy may be recommended for worsening renal function, and hemorrhage in the lungs and brain refractory to steroids and immunosuppressive drugs. High-dose IV pulse steroids, immunosuppression, plasmapheresis or intravenous immunoglobulin therapies are grade D recomendations based on meta-analytic studies [12]. 

There are some case reports showing that dapsone [1] or colchicine [23] may be useful in chronic HSP. Since Factor XIII levels are found to be low in HSP patients and correlate with the severity of gastrointestinal symptoms, Factor XIII replacement has been advocated as an adjunctive therapy [24].

### 2.8. Prognostic Factors

In general, most of the HSP cases are self-limited, with good prognosis and five-year survival rates of 95% [1, 2, 19–22]. One third of the patients have relapses, which are milder and shorter in duration, usually within 4 months, and involving the same organs [1]. The prognosis depends on the age of onset, extent of renal involvement and its course, extent of skin involvement, particularly above the waist line, immunoglobulin imbalance, and neurological involvement (Table 4). In adults, a 75% survival rate seen at 14.8 years may not be solely related to intrinsic HSP, as there may be other comorbid conditions.

### 2.9. Conclusion

Although HSP is uncommon in the adolescent age group, non-thrombocytopenic palpable purpura with multiorgan involvement (gastrointestinal, kidney and joints) should make one consider the diagnosis. Prompt diagnosis and multidisciplinary intervention can lead to appropriate management and mitigate potential complications, as illustrated in this case.

## Figures and Tables

**Table 1:** Etiology associations with HSP [1–3].

Bacterial:	Drugs:
Group A beta hemolytic	Quinolones
Streptococci	
Staphylococcus aureus	Clarithromycin
Helicobacter pylori	Acetaminophen
Mycoplasma	Codeine
	Etanercept
Viral:	
Hepatitis A	Tumors:
Hepatitis B	Non-small cell lung cancers
Hepatitis E	Prostate cancer
Herpes simplex	Lymphoma
Human parvovirus B19	Multiple myeloma
Varicella	
Adenovirus	Genetic:
CMV	Alpha-1 antitrypsin deficiency
HIV	Familial Mediteranean Fever
	HLA-DRB1*01
Vaccinations	HLA-B35
MMR (mumps, measles	
and rubella)	
Pneumococcal	Parasites:
Influenza	Toxocara canis
Meningococcal	
Hepatitis B	

*This list is not a comprehensive list.

**Table 2:** Diagnostic criteria of HSP (ACR and EuLAR & PReS) [8, 9].

EuLAR/PReS criteria—2006 [3]	American College of Rheumatology criteria—1990 [4]
Mandatory criterion:	
(i) Palpable purpura	Three or more of the following criteria are needed:
Plus at least one of the following criteria**:**	
(1) Diffuse abdominal pain	(1) Age 20 years or less at disease onset
(2) IgA deposition in any biopsy	(2) Palpable purpura
(3) Arthritis/arthralgias	(3) Acute abdominal pain with gastrointestinal bleeding
(4) Renal involvement (hematuria and/or proteinuria)	(4) Biopsy showing granulocytes in the walls of small arterioles or venules in superficial layers of skin

**Table 3:** Treatment of HSP, indications for different medications: [1, 10–12].

Medications	Indication	Comments
Acetaminophen, NSAIDs	Mild rash, arthritis	
Oral steroids (1-2 mg/Kg)	Severe rash, cutaneous edema, severe colicky abdominal pain, scrotal and testicular involvement	These cannot prevent development of systemic involvement but can be helpful for symptomatic treatment. These decrease the duration of symptoms when compared to placebo group
IV steroids (1-2 mg/Kg)	Same as oral steroids, should be given if patient is not able to tolerate oral medications	Same as oral steroids
High-dose IV pulse steroids	Nephrotic range proteinuria	Decreases ESRD progression (in some case series and reports)
High-dose IV pulse steroids plus immunosuppression	Rapidly progressive glomerulonephritis (RPGN), hemorrhagic involvement of lungs, brain	Grade D recommendation
Plasmapheresis and/or IV immunoglobulin therapy	Refractory HSP to combination therapy (steroids and immunosuppression), massive hemorrhage in gastrointestinal or other organs	Grade D recommendation, but evidence is growing with multiple case series and reports. This is used as the last resort to treat refractory HSP.

**Table 4:** Prognostic factors for HSP: [1, 2, 19–22].

The worse prognostic factors:
(i) Greater than 8 years of age
(ii) Greater number of relapses
(iii) Higher creatinine level at the onset
(iv) Proteinuria greater than 1 g/day
(v) Hematuria, anemia at diagnosis
(vi) Development of hypertension
(vii) Membranoproliferative glomerulonephritis
(viii) Fever at presentation
(ix) Purpura above the waist
(x) Persistent purpura
(xi) Elevated sedimentation rate.
(xii) Elevated IgA concentration with reduced IgM
concentration at the time of diagnosis.
(xiii) Low factor XIII level
